# Humidity Sensors Based on Cellulose Nanofiber Fabricated on a Three-Dimensional (3D) Curved Surface

**DOI:** 10.3390/nano13233005

**Published:** 2023-11-23

**Authors:** Mijin Won, Gyeongseok Oh, Hyunah Lee, Jaehwan Kim, Dong-Soo Kim

**Affiliations:** 1 Department of Creative Convergence Engineering, Hanbat National University, Yuseong-ku, Daejeon 34158, Republic of Korea; 0mj00@naver.com (M.W.);; 2 Creative Research Center for Nanocellulose Future Composites, Inha University, Incheon 22212, Republic of Korea; jaehwan@inha.ac.kr

**Keywords:** reverse offset, double-layer blanket, 3D curved surface, humidity sensor, 3D-printed electronics

## Abstract

Traditional printed electronics processes have recently been utilized within 3D-printed structures where components and interconnects are introduced during manufacturing disruptions. The dielectric performance of 3D-printed materials has a low-resolution problem, and many technologies have been proposed for direct printing on a 3D curved surface or structure. This paper reports a humidity sensor fabricated with a 3D-printed electrode and cellulose nanofibers on a curved surface. The electrode part of an interdigital electrode (IDE) sensor is printed on a flat glass substrate and a 3D-curved glass substrate using a double blanket reverse offset. Subsequently, a cellulose nanofiber emulsion is coated onto the IDE pattern as a sensing layer with a dispenser. The electrical impedance of the sensor is measured with the relative humidity (RH) changes between 10% and 90% RH. The sensor demonstrates a high repeatability and sensitivity, even on a 3D curved substrate. This technology provides a promising method to integrate humidity sensors and 3D deformable surfaces.

## 1. Introduction

Humidity sensors are used in various fields, including environmental monitoring, automobiles, and medical care. Different types of humidity sensors that operate on different transduction principles exist, such as resistive, capacitive, optical, and surface elastic wave types [1,2,3,4]. Sensors based on the impedance and capacitance responses are the most appropriate for humidity sensing owing to their low cost, ease of fabrication, and nominal time response, as well as the ability to detect the moisture content of the environment using variables such as resistance and capacitance [5,6,7,8,9,10]. Resistive-type sensors are less expensive and easier to read compared to other sensors.

In general, rigid substrates, such as ceramics, glass, and silicone, are used to fabricate sensors. Flexible and deformable printed sensors have become popular since they offer a cost-effective and versatile alternative to traditional rigid sensors. The ability to print sensor devices onto various shapes and substrates, including plastics, textiles, and paper, has opened new avenues for innovation in humidity sensors. Recently, research has been conducted on insertable devices that can be mounted on light, flexible, and thin substrates using printing [11]. Chemosensitive materials are important in determining humidity-sensor performance, and polymeric chemosensitive materials are beneficial for flexible humidity sensors. Various polymeric materials are adopted for humidity sensors: polyethyleneimine, dibromoethane, sodium polystyrenesulfonate, polyacetylenes, ethylated polymers, poly(chloromethyl styrene), epoxy resin containing quaternary ammonium salts, and cellulose [12].

For resistance-type sensors, extensive research is being conducted on the printing process, in which humidity sensors are manufactured using various printing techniques on diverse substrates, including rigid substrates. Most electronic devices and sensors are made on 2D surfaces, robust and brittle substrates suitable for various applications in diverse fields. However, they are incompatible with complex curved surfaces, such as soft 3D living organisms in the health care/monitoring areas or irregular curved wings required for equilateral integration [13]. Thus, we should be able to fabricate humidity sensors on 3D surfaces.

The measurement accuracy and sensitivity of existing sensors are limited at high temperatures or humidity. As a result, it is very important to implement a humidity sensor with high sensitivity, flexibility, and a wide response range for integrating it with existing sensors. In particular, a specific range can be set, and a humidity sensor can be manufactured with patterned electrodes on a curved surface using various printing methods. In printing humidity sensors, fine-line printing capabilities are required.

In this study, the proposed double blanket reverse offset (DB-RO) printing can print fine patterns with a line width of several micrometers on a 3D substrate. DB-RO printing can realize fine line widths without causing damage to the substrate using a soft blanket. It has the advantages of a high resolution and the precision of traditional lithography, which can be a key in fabricating precise and deformable curved sensors. Therefore, the humidity sensor is manufactured in this study using DB-RO on a curved surface, demonstrating a soft blanket hardness, enabling 3D printing.

Printing several materials with a single process remains limited and is a major issue associated with producing a curved humidity sensor using an inkjet and dispenser, which are conventional noncontact printing methods. It is not easy to realize a complete sensor device using these methods. When a sensor device is manufactured directly on a 3D curved surface, the structure control range of the device is widened, which enables the device fabrication with high functionality. Curved lithography can be applied to all surfaces with a high pattern resolution, but the challenges are deposition, etching, or material growth/doping in a wide area of the curved surfaces [14,15]. Thus, when the accuracy requirement is not high or the device structure is not complicated, it is ideal for producing devices by integrating them on any surface through equilateral inkjet printing, 3D printing, or laser direct writing.

Ideal humidity-sensor materials require high sensitivities, wide humidity-sensing ranges, fast response times, short recovery times, and inexpensive manufacturing methods [15]. Herein, we used cellulose nanofiber (CNF) as the reactant for the humidity sensor. Cellulose, the most abundant polymer in nature, is inexpensive, easy to process, renewable, easy to modify chemically, mechanically strong, and has smart sensing and actuating behaviors. [16,17,18]. Moreover, CNF demonstrates low humidity hysteresis, excellent linearity, and fast response recovery [19,20]. CNFs have been utilized for humidity sensors by blending with carbon nanotubes and graphene nanoplatelets [21,22,23,24]. Based on these characteristics of CNF, flexible, biodegradable, and inexpensive sensors can be designed.

**Figure 1:**
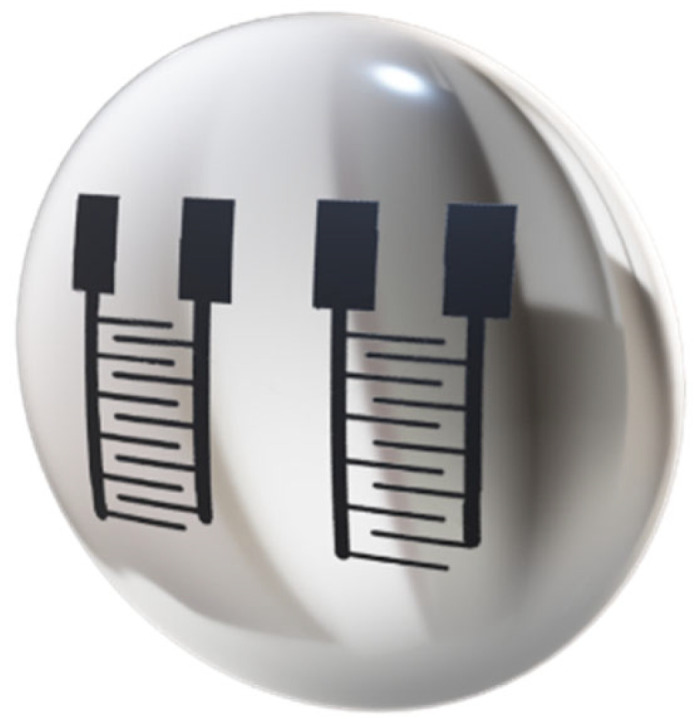
Schematic model of the impedance-type 3D humidity sensor.

This study aims to manufacture humidity sensors on a 3D curved substrate through a printing process equipped with a double blanket. An interdigital electrode (IDE) pattern with a line width of 40 µm and a width of 40 µm is fabricated using DB-RO equipment to obtain signals from the humidity sensors. A CNF sensing layer is deposited on the IDE pattern by dispensing CNF reactant. The humidity-sensing behavior of the sensor fabricated using the simple and direct printing process on the 3D curved substrate is investigated. Figure 1 shows the schematic of the humidity sensor prepared on a spherical surface.

## 2. Experiment

### 2.1. Materials

Silver conductive ink (viscosity: 1.5 cps, surface tension: 24.4 mN/m, dispersion matrix: octane-based) was purchased from ANP in Chungcheongbuk-do, Republic of Korea. A PDMS (polydimethylsiloxane)-based blanket (#CF 0.70) was purchased from Fujikura Composites, Tokyo, Japan. A glass substrate was used as the substrate, and a flat glass with a radius of curvature of 10° was used. The CNF suspension was isolated from a hardwood pulp (bleached acacia kraft pulp, Hansol paper, South Korea) by using a 2,2,6,6-tetramethylpiperidine-1-oxyl (TEMPO) oxidation and aqueous counter collision (ACC) method [21]. All chemicals were purchased from Sigma-Aldrich (Burlington, MA, USA).

### 2.2. Sensor Fabrication

The humidity sensor comprises an IDE pattern fabricated on a 3D curved substrate and coating a CNF sensing layer. The sensing mechanism of the humidity sensor is based on resistance conversion, and the electrical conductivity of the sensing layer increases due to the absorption/desorption of water molecules by the sensitive CNF layer. CNFs have many hydroxyl groups on their surface, and two water molecules exist: adsorbed water and free water molecules. Adsorbed water molecules bind CNFs tightly by making hydrogen bonds, forming a solid CNF layer. On the other hand, free water molecules can be easily absorbed/desorbed to CNFs depending on the environment humidity. Depending on the amount of adsorbed free water molecules, the electrical resistance of the CNF layer can be changed, giving it its humidity-sensing behavior.

**Figure 2:**
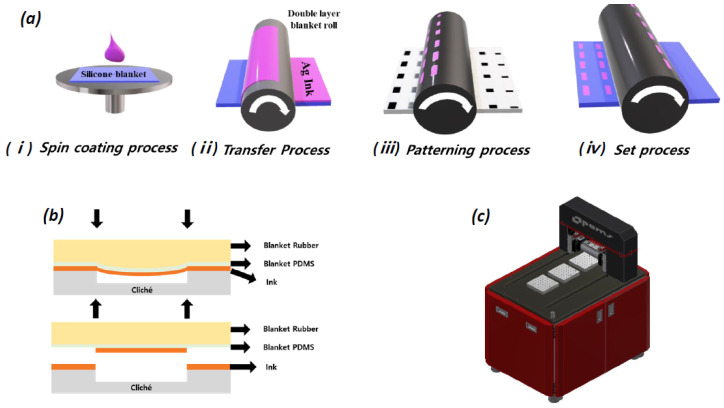
(**a**) Steps involved in the fabrication process, (**b**) principle of double blanket transfer, and (**c**) DB-RO equipment.

The change in conductivity based on the humidity level can be easily quantified by measuring the resistance between the IDEs. The stepwise fabrication of IDEs is shown in Figure 2, where silver ink is used for the electrode fabrication. High-resolution bottom electrodes were fabricated on a 3D shape using a DB-RO printing system (PEMS, Reverse offset, Gyeonggi-do, Republic of Korea). The IDE pattern has 100 finger pairs with a 40 µm finger gap. At first, a silicon blanket was made on a glass substrate by spin coating the PDMS, and the silver ink was coated at 4000 rpm, 20 s on the silicone blanket (Figure 2(ai)). After attaching the silicone blanket to the reverse offset device, the ink was transferred to the roll blanket at 3 mm/s under 1 kg_f_ double blanket pressure (Figure 2(aii)). After the ink was transferred to the roll blanket, the pattern was turned off in the cliché where the pattern was inscribed. The pattern transfer to the roll blanket was printed on a 3D substrate. Figure 2b represents how the ink is transferred to cliché, and Figure 2c shows the DB-RO printing system.

A dispenser technology capable of thin film coating was used to deposit the CNF sensing layer. The cellulose reactant used in the sensing layer was a CNF suspension, and it was diluted with DI water in a ratio of 1:2. The hardwood CNF width and length are 15.1 ± 5.2 nm and 652 ± 263 nm, respectively [25]. The crystallinity index is about 71.7%, and the carboxyl content was 0.67 mmol/g [25].

The manufacturing process of the proposed humidity sensor is fast and compatible with roll-to-roll technology. Furthermore, unlike semiconductor manufacturing, the proposed fabrication process is cost effective and does not require high temperatures [26].

### 2.3. Sensor Characterization and Humidity Measurement

**Figure 3:**
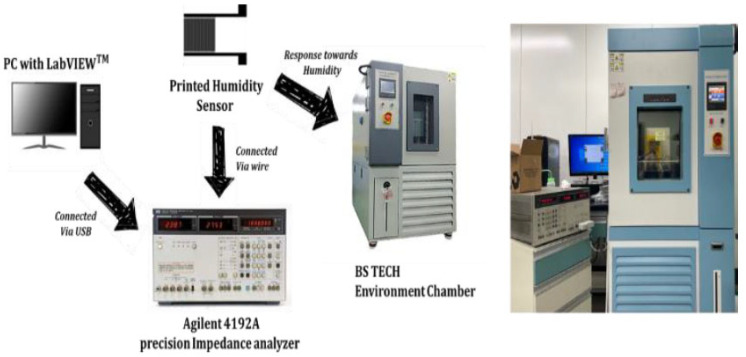
Schematic diagram of the humidity-measurement setup showing the individual components.

The morphologies of the CNF sensing layer were characterized using an SEM (S-4800, Hitachi, Tokyo, Japan) at an accelerating voltage of 15 kV after coating 3 nm of platinum. Ag IDEs were characterized via the SEM and a 3D profiler system (NV-2400, Nano System, Daejeon, Republic of Korea). The manufactured humidity sensor was obtained via real-time monitoring of the temperature and relative humidity (RH) at room temperature inside an environment chamber (TEMP&HUMID CHAMBER, BS Tech, Gyeonggi, Republic of Korea). The humidity-sensing response was recorded as a change in the resistance with the RH. An impedance analyzer (Agilent 4192A, Santa Clara, CA, USA) powered by a custom-designed Labview-based interface monitored the resistance across the IDE in real time (Figure 3). The input voltage of the impedance measurement was 1V, and the frequency was fixed at 1 kHz [27].

## 3. Results and Discussion

### 3.1. Sensor Characterization

**Figure 4:**
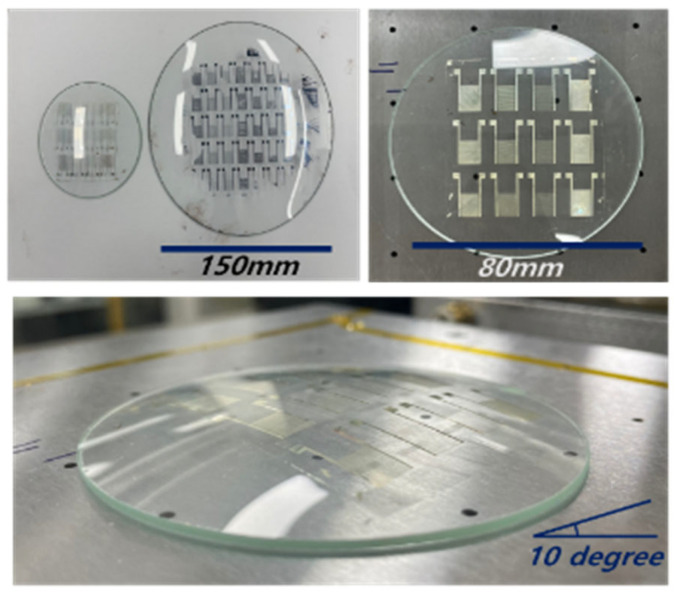
Photographs of the fabricated humidity-sensor array printed on a 3D curved structure.

**Figure 5:**
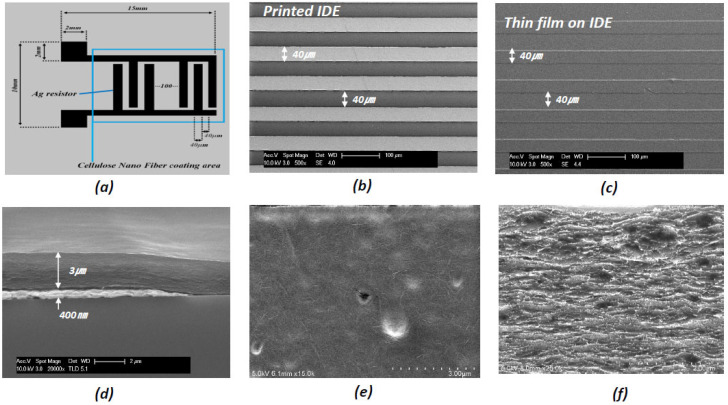
(**a**) Schematic model of the humidity sensor, (**b**) SEM image of the fabricated IDE, (**c**) SEM image of the CNF coating layer over the IDE, (**d**) cross section of CNF layer, and (**e**,**f**) surface and cross section SEMs of the CNF sensing layer.

The large-scale fabrication of the humidity-sensor array was successfully achieved using the proposed process. Figure 4 shows photographs of the humidity-sensor array comprising 36 units with an overall 100 × 100 mm^2^ dimension. Ag IDEs can be formed rapidly and easily using a printing system. The total sensing area is 15 mm × 10 mm. Figure 5a shows the IDE dimension of the humidity sensor. The sensor line width produced by printing was 30 µm, and the finger-to-finger spacing was 40 µm, realizing a fine line width, as shown in Figure 5b–c. In addition, the sensors printed on the 3D curved surface were finely printed. The flat and 3D substrates were printed with the same line width. While printing on the curved substrate, the roll pressure cannot be excluded. The DB-RO process could print the pattern on the curved substrate without collapsing. This result indicates that the hardness of the blanket was lower than that of a general blanket roll. Thus, printing on the curved surface was successfully achieved.

**Figure 6:**
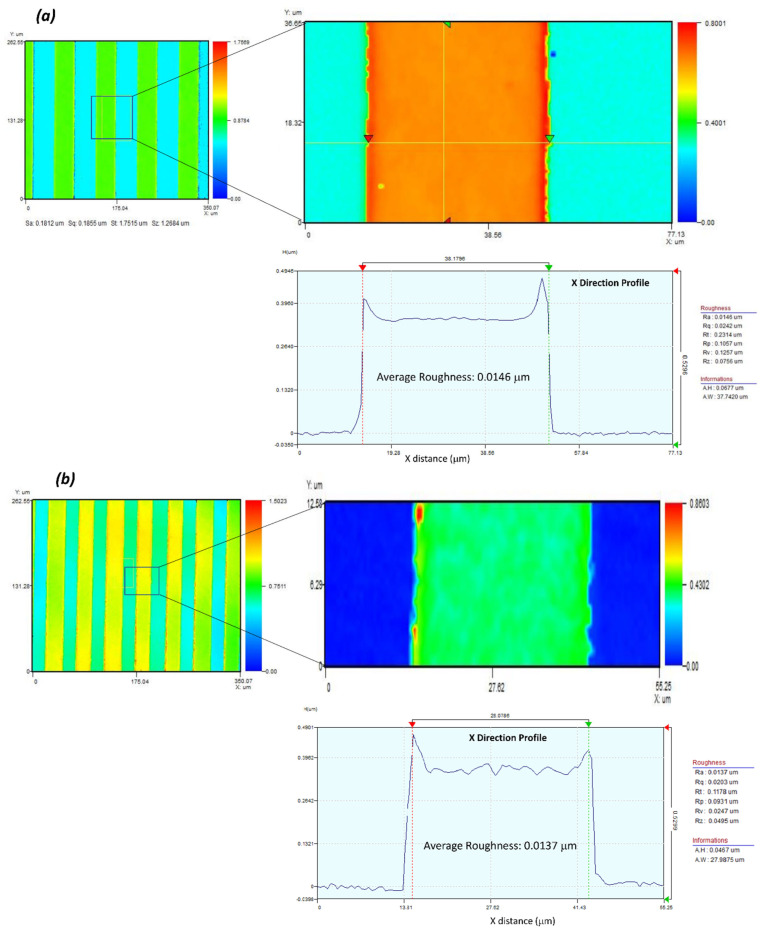
The 3D output of the vertical scanning interferometry showing average roughness: (**a**) the flat glass substrate and (**b**) the curved glass substrate.

The CNF-layer thickness can be controlled by the dispenser’s air pressure. Figure 5d shows the cross-sectional SEM image of the electrode and sensing layers, showing the IDE layer’s thickness is 400 nm and the CNF sensing layer’s thickness is approximately 3 µm. Figure 5e,f show the surface and cross-sectional SEM images of the CNF sensing layer, indicating compact and smooth-packing CNFs. Figure 6 represents the surface profiles of the IDE patterns fabricated on (a) a flat glass and (b) the curved glass substrate. The surface roughness of the curved glass substrate, 13.7 nm, is nearly the same as the flat glass substrate result (14.6 nm), indicating the 3D-printed IDE pattern on the curved substrate was successfully made.

### 3.2. Humidity-Sensor Testing

The humidity sensing of the fabricated sensor was tested at different RH values ranging between 10 and 90% RH. The sensitivity, repeatability, hysteresis, and response time were evaluated.

#### 3.2.1. Sensitivity and Linearity

**Figure 7:**
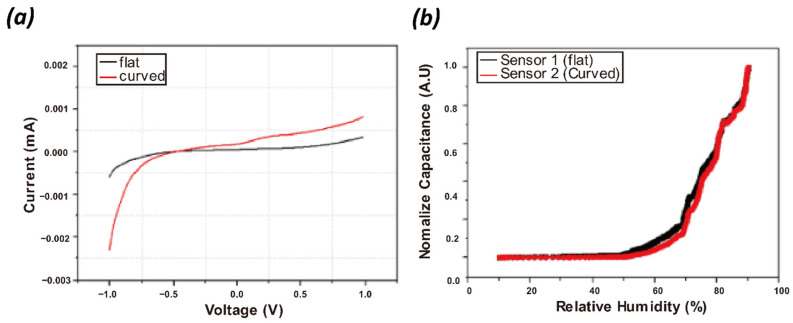
(**a**) Typical I–V curves of humidity sensors fabricated on flat and curved surfaces and (**b**) normalized capacitance values with the RH change.

The humidity sensitivity of the sensor fabricated on the curved substrate was compared with the flat substrate sensor, and Figure 7a represents their current–voltage (I–V) curves. They exhibit linear I–V characteristics, and the shape of the substrate resulted in a slight change in the slope of the curve. Figure 7b shows the normalized capacitance of the sensors with the RH change. As the RH increases, the sensor responses linearly increase up to 60 RH% and steeply increase after that, indicating that the CNF sensing layer can sense the humidity change effectively.

#### 3.2.2. Hysteresis, Response Time, and Stability

**Figure 8:**
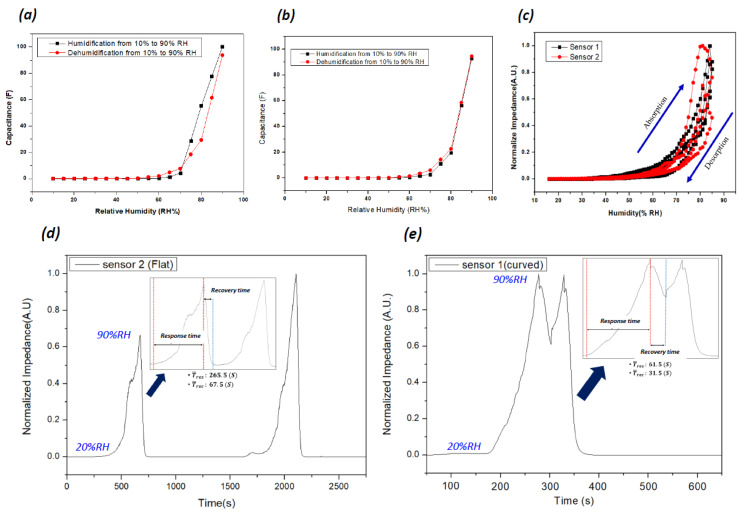
Impedance response hysteresis curves of (**a**) sensor 1 (flat substrate), (**b**) sensor 2 (curved substrate), and (**c**) one-cycle hysteresis showing absorption and desorption from RH 20–90% at 1 kHz. (**d**,**e**) Response values of the sensors based on a flat CNF substrate and curved substrate.

The patterned sensor can easily be transferred to arbitrarily shaped substrates without causing any damage. The CNF sensing layer on the IDE, after being transferred to a curved surface, is shown in Figure 4, where the water-molecule absorption changes the dielectric property of the CNF layer, resulting in the capacitance change. Due to the hydroxyl groups of CNF, water molecules can easily interact with the CNF surface. Water-molecule adsorption is associated with hysteresis and response time. Thus, the humidity sensors were tested by increasing and decreasing the humidity between 10% RH and 90% RH. Figure 8a–b show the capacitance changes of the humidity sensor on the flat substrate and the curved substrate, respectively, with the humidity change. The capacitance values slowly increase linearly with the increase of the RH up to 65% and ramp up after that. Note that the curved humidity sensor shows less hysteresis than the flat one. Figure 8c illustrates one cycle of hysteresis in the normalized capacitance scale, showing the adsorption and desorption of humidity. The hydrophilic functional groups on CNF attract water molecules easily and increase the capacitances of sensors, and hysteresis occurs because of the different adsorption and desorption speeds of water molecules in the CNF layer.

Figure 8d,e show the response times of the sensors. The rising time of the flat sensor humidity sensor from 20% RH to 90% RH is 265.5 s, and the curved humidity sensor is 61.5 s. Also, their recovery times are 61.5 s and 31.5 s, respectively. The fast response time of the curved humidity sensor might be associated with the CNF-layer thickness; the CNF coating thickness was thicker on the flat substrate than on the curved substrate. The CNF suspension has a low viscosity, so it can spread on the curved surface more easily than the flat surface, resulting in a thinner coating on the curved substrate. Thus, the curved humidity sensor shows a fast response time and less hysteresis. The response and recovery rates of the sensors were similar to those obtained in the previous nanocellulose-based sensor studies.

**Figure 9:**
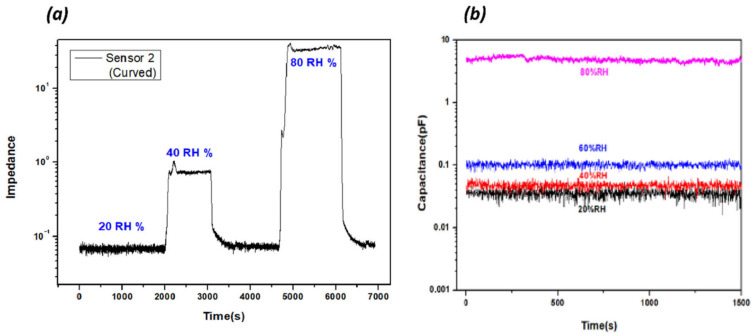
(**a**,**b**) Stability analysis for a curved humidity sensor at different %RH values.

Stability is an important factor for real applications of humidity sensors. The RH was maintained, and the sensor response was monitored to check the stability. Figure 9a,b show the stability of the curved humidity sensor with time under different RH levels. Slight fluctuations are shown in each RH level, but the fluctuations are very stable, indicating stable responses. The humidity sensor was manufactured using a direct coating process to a curved substrate. It indicates that various devices can be fabricated to 3D arbitrarily shaped substrates.

## 4. Conclusions

This study examined the challenges of fabricating curved humidity sensors with a 3D-printed electrode and cellulose nanofibers. The interdigital electrode (IDE) part of the 3D humidity sensor was manufactured using the DB-RO printing process with silver conductive ink. The printing process realized a fine line width with a high resolution. A CNF sensing layer was coated on the IDE to form the 3D humidity sensor. The same humidity sensor was fabricated on a flat substrate and compared with the curved humidity sensor. The CNF-based curved humidity sensor detected between 10% and 90% RH levels with low hysteresis.

Additionally, the curved sensor exhibited a very fast response and recovery times of 61.5 s and 31.5 s, respectively. The curved humidity sensor showed lower hysteresis and a shorter response time than the flat one. The sensor demonstrates a high repeatability and sensitivity, even on a 3D curved substrate. The proposed method provides a promising strategy for fabricating electronic and sensor devices on various substrates, including curved ones.

## Data Availability

Data are contained within the article.
